# *TPR5* is involved in directional cell division and is essential for the maintenance of meristem cell organization in *Arabidopsis thaliana*

**DOI:** 10.1093/jxb/erw043

**Published:** 2016-02-17

**Authors:** Naoyuki Sotta, Lukram Shantikumar, Takuya Sakamoto, Sachihiro Matsunaga, Toru Fujiwara

**Affiliations:** ^1^Department of Applied Biological Chemistry, Graduate School of Agricultural and Life Sciences, The University of Tokyo, Yayoi, Bunkyo-ku, Tokyo 113–8657, Japan; ^2^Department of Applied Biological Science, Faculty of Science and Technology, Tokyo University of Science, 2641 Yamazaki, Noda, Chiba 278–8510, Japan

**Keywords:** *Arabidopsis thaliana*, cell division, post-embryonic development, root growth, root radial structure, TPR protein.

## Abstract

Root growth in plants is achieved through the co-ordination of cell division and expansion. In higher plants, the radial structure of the roots is formed during embryogenesis and maintained thereafter throughout development. Here we show that the tetratricopeptide repeat domain protein *TPR5* is necessary for maintaining radial structure and growth rates in *Arabidopsis thaliana* roots. We isolated an *A. thaliana* mutant with reduced root growth and determined that *TPR5* was the gene responsible for the phenotype. The root growth rate of the *tpr5-1* mutant was reduced to ~60% of that in wild-type plants. The radial structure was disturbed by the occurrence of occasional extra periclinal cell divisions. While the number of meristematic cells was reduced in the *tpr5* mutants, the cell length in the mature portion of the root did not differ from that of the wild type, suggesting that TPR5 is required for proper cell division but dispensable for cell elongation. Expression of the TPR5–GFP fusion protein driven by the *TPR5* promoter displayed fluorescence in the cytoplasm of root meristems, but not in mature root regions. DNA staining revealed that frequencies of micronuclei were increased in root meristems of *tpr5* mutants. From this study, we concluded that *TPR5* is involved in preventing the formation of micronuclei and is necessary for both the activity and directionality of cell division in root meristems.

## Highlight

*Arabidopsis thaliana TPR5* is involved in proper alignment of the cell division plane and root elongation and is required for preventing micronuclei formation.

## Introduction

The roots of *Arabidopsis thaliana* display radial cellular organization arranged in the order of stele, endodermis, cortex, and epidermis cells from the inside to the outside (Dolan *et al.*, 1993). The fundamental structure is conserved through the roots, enabling continuous root elongation and efficient substance transport. Root elongation is achieved both by cell elongation in the elongation zone and cell proliferation in proximal meristems. The size of the proximal meristem is conserved during post-embryonic development via a balance between cell proliferation and differentiation (Beemster and Baskin, 1998). In root meristems, the quiescent centre (QC) renders the surrounding cells as stem cells and forms a stem cell cluster which is called the stem cell niche and includes the stele initials, pericycle initials, cortex/endodermis initials (CEI), and epidermis/lateral root cap initials (van den Berg *et al.*, 1997). The stem cells undergo asymmetric cell division to produce self-renewing cells and daughter cells (Dolan *et al.*, 1993). Whereas daughter cells derived from stele and pericycle initials undergo symmetric cell division, CEI daughter cells divide asymmetrically resulting in a cortex cell and endodermal cell couplet. Similarly, the epidermis/lateral root cap initial daughter cells divide into epidermal cells and lateral root cap cells. These daughter cells detach from stem cell niches and undergo several rounds of symmetric, longitudinal division before migrating from the proximal meristems to the transition zone and losing their cell division activity (see review by Perilli *et al.*, 2012). During cell proliferation, it is critical to maintain the cell division planes perpendicular to the elongating axis; otherwise, the radial structure will not be maintained.

Several genes are involved in the proper alignment of the cell division plane and mutation of diverse genes involved in the establishment of the division site results in mis-positioned cell plates (see review by Müller, 2012). *TONNEAU2*/*FASS* is a putative regulator of protein phosphatase A2, which is necessary for pre-prophase band (PPB) assembly and its mutants exhibit cell division planes in random orientations (Traas *et al.*, 1995; Camilleri *et al.*, 2002). *TONNEAU1* interacts with centrin and is essential for PPB formation (Azimzadeh *et al.*, 2008). TANGLED and RanGAP, whose depletion results in disorganized root cell files, is concentrated at the PPB and remains associated with the cortical division site (Walker *et al.*, 2007; Xu *et al.*, 2008). The mitogen-activated protein kinase MPK6, which is localized in the PPB and phragmoplast, is involved in control of the cell division plane during early development (Müller *et al.*, 2010). Mutants of PHRAGMOPLAST ORIENTING KINESIN 1 and 2 exhibit improper placement of cell walls (Müller *et al.*, 2006). Although these extensive studies have revealed the mechanisms of cell division plane alignment (see review by Müller, 2012), gaps in our understanding of these complex mechanisms remain.

Here we report the identification and characterization of a novel player in *Arabidopsis thaliana* root patterning, the tetratricopeptide repeat (TPR) domain protein TPR5. While the TPR domain is known to interact with other proteins to form complexes (Lamb *et al.*, 1995), to our knowledge, there have been no studies on the involvement of *TPR5* in any biological processes. We demonstrated that *tpr5* mutants exhibited slower root elongation, disordered radial root cell organization with misplaced cell division planes, and decreased numbers of meristematic cells. In addition, we demonstrated that *TPR5* is expressed in root meristems throughout the cell cycle and is necessary for preventing micronuclei formation.

## Materials and methods

### Plant materials and growth conditions

The B13.4/*tpr5-1* mutant was selected from a Col-0 *gl1-1* ethylmethane sulphonate irradiated M_2_ population (Lehle seeds, USA), and *tpr5-2* (SALK099949) was obtained from the Arabidopsis Biological Resource Center. The T-DNA homozygous line was established using the PCR primers SALK099949_LP and SALK099949_RP (see Supplementary Table S1 at *JXB* online).

Wild-type (Col-0) or mutant seeds were surface-sterilized for 1min with 70% ethanol and for 1min with 99% ethanol. After removing the ethanol, the seeds were sown on sterilized MGRL media (Fujiwara *et al.*, 1992) plates supplemented with 1% sucrose, solidified with 1.5% gellan gum, and then incubated at 4 °C for 2 d. Plates were placed vertically in incubators at 22 °C under a 16/8h light/dark cycle.

### Reverse transcription-polymerase chain reaction (RT-PCR)

Total RNA was prepared from whole roots of 15-d-old seedlings with the RNeasy Plant Mini Kit (Qiagen) according to the manufacturer’s instructions. Approximately 500ng total RNA were used for reverse transcription with Prime Script RT Master Mix (Takara, Japan) according to the instructions using the 10 µl scale. The product was diluted 10-fold and used as PCR templates. For semi-quantitative RT-PCR, the *TPR5* coding sequence and *Actin8* were amplified by three-step PCR with Go taq Green Master Mix (Promega). The PCR conditions were as follows: denaturation at 95 °C for 2min, then 31 cycles of 95 °C for 30s, 55 °C for 30s, and 72 °C for 20s (90s for *TPR5*), followed by extension at 72 °C for 7min. Primer sets TPR5_CDS_F (and _R) or ACTIN8_RT_F (and _R) were used (Supplementary Table S1). Quantitative RT-PCR of *CYCB1;1* was performed with SYBR^®^ *Premix Ex Taq*™ II (Tli RNaseH Plus, Takara) based on the protocol provided by the manufacturer. *Actin8* was used as an internal control and primer sets CYCB1;1_RT_F (and _R) or ACTIN8_F (and _R) were used (Supplementary Table S1).

### Positional identification of the responsible gene

For genetic linkage analysis, the F_2_ generation was obtained from a cross between B13.4 (Col-0 background) and L*er*. Single sequence length polymorphism (SSLP) and single nucleotide polymorphism (SNP) markers between Col-0 and L*er* were used to detect the genotype. Genetic markers near the candidate region are shown in Supplementary Table S2.

### Root length measurement and counting of lateral root numbers

Seedlings on medium plates were photographed using a Canon EOS Kiss digital camera and images were saved using JPEG. The root length was measured from the digital images using the segmented line mode of the ImageJ software (http://rsbweb.nih.gov/ij/). The number of emerged lateral roots was counted via observation under a stereomicroscope.

### Generation of transgenic plants

For the complementation test, either genomic or coding DNA sequences (CDS) were introduced into *tpr5-2*. For the genomic sequence line, the promoter region and open reading frame excluding the stop codon were amplified from the genomic sequence by PCR with the primers TRP5_pro_F and TPR5_CDS_R. For the CDS line, the coding sequence excluding the stop codon was amplified from cDNA using TPR5_fuse_F and TPR5_CDS_R primers. The promoter region was amplified with primer TPR5_pro_F and TPR5_fuse_R. These fragments were mixed and fused using PCR and amplified with the TRP5_pro_F and TPR5_CDS_R primers. The DNA fragments were introduced into pENTR / D-TOPO vector (Invitrogen) and transferred into pMDC107 vector (Curtis and Grossniklaus, 2003) using the gateway LR clonase recombination system (Invitrogen).

To generate the promoter–GUS line, the promoter region of *TPR5* was cloned into the pENTR/D-TOPO vector using primers TPR5_pro_F and TPR5_pro_R and transferred into the pMDC162 vector (Curtis and Grossniklaus, 2003) using the gateway LR clonase recombination system.

The binary vectors were introduced into *Agrobacterium tumefaciens* strain GV3101 (Bechtold and Pelletier, 1998) and *tpr5-2* (for the complementation test) or Col-0 (for promoter–GUS analysis) plants were transformed with these cultures using the floral dipping method (Clough and Bent, 1998). The transformed plants were selected on half-strength Murashige and Skoog (MS) medium containing 20 µg ml^–1^ hygromycin B (Wako) and 250 µg ml^–1^ Claforan (Sanofi, Japan), solidified with 0.5% agarose.

### GUS staining

Seedlings were vacuum infiltrated with GUS staining solution comprising 100mM Na_2_HPO_4_ buffer pH 7.0, 0.1% Triton X-100, 2mM K_3_Fe[CN]_6_, K_4_Fe[CN]_6,_ and 0.5mg ml^−1^ X-GlcA (5-bromo-4-chloro-3-indolyl-β-d-glucuronide cyclohexyl ammonium salt, Wako, Japan) for 15min at room temperature and incubated at 37 °C in the dark for 16h. Whole seedlings were photographed using a Canon Eos Kiss digital camera. For detailed observation by microscopy, stained seedlings were clarified by overnight incubation with chloral hydrate solution (4g chloral hydrate, 1ml glycerol, and 2ml water) on microscope slides and observed under an optical microscope with bright field or differential interference contrast.

### Confocal microscopy

For observation of the cell wall, roots were cut and mounted on a slide glass with 10 μg ml^–1^ PI solution and observed after 15min. Fluorescence from PI was observed using a confocal laser scanning microscope FV1000 or FV1200 (Olympus). The wavelengths for excitation and emission were 559nm and 570–670nm, respectively. For observation of GFP fluorescence of transgenic plants, roots were mounted with water and observed using 473nm and 510nm for excitation and emission, respectively. For DNA staining, roots were fixed in 4% (w/v) formaldehyde in PBS (137mM NaCl, 2.68mM KCl, 8.1mM Na_2_HPO_4_, and·1.47mM KH_2_PO_4_) for 10min at 4 °C. After washing with PBS, the fixed roots were stained with 4′,6-diamidino-2-phenylindole (DAPI) using the staining buffer CyStain^®^ UV Precise P DNA staining kit for 2min at room temperature. Stained samples were washed and mounted with PBS. The DAPI signal was detected using confocal microscopy with wavelengths of 405nm and 461nm for excitation and emission, respectively.

### Assessment of the cell cycle stages

For the detection of cells in the S phase, seedlings 3 d after germination (DAG) were placed in liquid medium containing half-strength-MGRL and 10 μM 5-ethynyl-2′-deoxyuridine (EdU) in Click-iT component A (Invitrogen) for 30min at 22 °C under continuous light. EdU incorporation was stopped by fixation with 4% PFA/PBS for 30min under vacuum. After three washes with PBS, the seedlings were incubated with 0.5% Triton X-100/PBS for 20min. After three washes with PBS, EdU was labelled with Alexa Fluor 594 azide following the manufacturer’s instructions. Nuclei were stained with SYBR Green I (Lonza) diluted 5 000-fold with 0.5% Triton X-100/PBS for 10min. The seedlings were then mounted with 1/2× mounting medium as described in Hayashi *et al.* (2013). Fluorescence emitted from Alexa Fluor 594 and SYBR Green I was observed using a fluorescent microscope (IX-81, Olympus) equipped with a confocal scanning unit (CSUX-1, Yokogawa) and a sCMOS camera (Neo 5.5 sCMOS ANDOR Technology). The excitation and emission wavelengths were 561nm and 604–644nm for EdU and 488nm and 503–537nm for SYBR Green I, respectively. Images were analysed using ImageJ software. M phase cells were distinguished from other cells, based on SYBR Green I staining, with obvious features of prophase, metaphase, anaphase, and telophase.

## Results

### Slow root elongation and small shoots of the B13.4 mutant

**Fig. 1.**
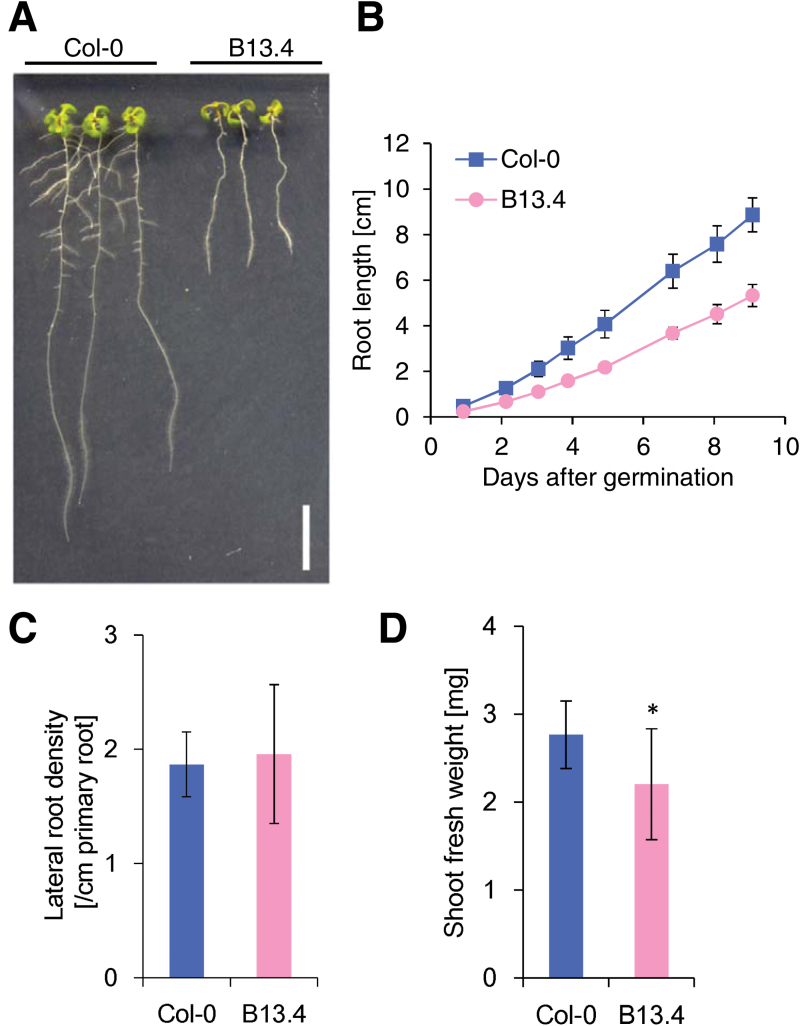
Growth of the B13.4 mutant. (A) Seven DAG seedlings from the wild type and B13.4 mutant grown on MGRL plates. Bar, 1cm. (B) Time-course of root length-change in the wild type and B13.4 mutant after germination. Values represent the mean ±standard deviations of 14–19 measurements. (C) Lateral root density per primary root length. Lateral root number of 7 DAG seedlings was counted using stereomicroscopy. No significant difference was detected between Col-0 and B13.4 by Welch’s *t* test at *P* <0.05. *n*=15–16 seedlings. (D) Shoot fresh weight measurement of the 7 DAG wild type and B13.4 mutant. Values represent the mean ±standard deviations of 11–20 measurements. Asterisks indicate a significant difference from Col-0 at *P* <0.05 by Welch’s *t* test.

We isolated an *A. thaliana* mutant by screening for mutants defective in root growth. The phenotype was confirmed in the M_3_ generation and the mutant line was termed B13.4 (Fig. 1A). B13.4 carried a single recessive mutation responsible for the phenotype. To characterize the root growth of B13.4 in detail, we measured the root length of the mutant almost every day up to 9 DAG. The primary root length of B13.4 was about two-thirds that of the wild type throughout the growth period (Fig. 1B). Assuming constant growth rates of the roots, the average growth rate of B13.4 was 61% of that in the wild type. Although B13.4 appeared to develop fewer lateral roots at 7 DAG (Fig. 1A), microscopic observation revealed that the number of emerged lateral roots per primary root length did not differ significantly between the wild type and B13.4 (Fig. 1C). B13.4 exhibited smaller shoots compared with the wild type (Fig. 1D)

### Decreased cell number in B13.4 root meristems

**Fig. 2.**
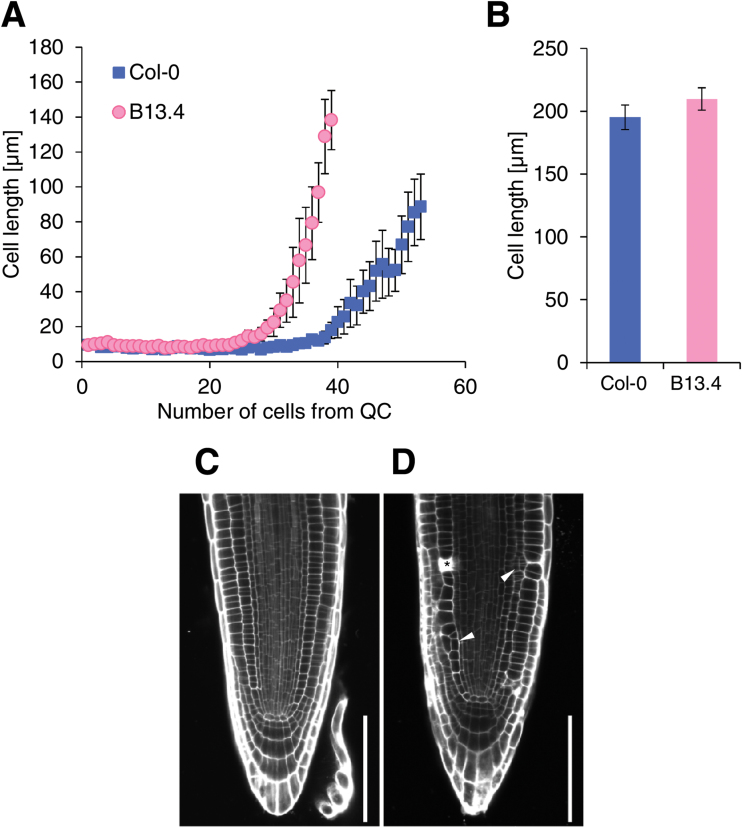
Root cell organization of B13.4. (A) Longitudinal cell length of each cortical cell in root meristems of 3 DAG seedlings. Cell numbers were counted from the quiescent centre. Values are the mean ±standard error of measurements from 10 seedlings. (B) Longitudinal length of mature cortex cells in 3 DAG wild type and B13.4. Values represent the mean ±standard errors of at least 60 measurements from 10 individual seedlings. (C, D) Confocal images of 3 DAG wild-type (C) and B13.4 (D) roots stained with PI. Arrowheads indicate extra periclinal cell divisions, and an asterisk indicates dead cells stained with PI. Bars=100 µm.

The root growth rate was determined by meristem activity and cell expansion (Beemster *et al.*, 1998). To evaluate meristem activity, we estimated the cell number in root meristems. The meristematic zone was defined as the area between the QC and the first elongating cortical cell. To estimate the boundary between the meristem and the elongation zone quantitatively, we measured the lengths of all cortical cells from the QC to the elongation zone in 3 DAG seedlings. The data suggested that the average cell length starts to increase at the 24th and 35th cells from the QC in B13.4 and the wild type, respectively (Fig. 2A). The different positions of the initiation of cell elongation indicate that the number of meristematic cells was reduced in B13.4.

To compare the final lengths of the root cells, we measured the cell length in the mature region of B13.4 and wild-type roots. No significant difference was observed in mature cell length between B13.4 and wild-type roots (Fig. 2B).

### Perturbation in the radial structure and occasional cell death in B13.4

Propidium iodide (PI)-stained seedlings were observed at 3 DAG using a confocal microscope. The cellular organization in the root meristems differed between the wild type and B13.4 (Fig. 2C, D). In B13.4, the cell files in the root meristems were partially disordered with the occurrence of non-canonical periclinal cell divisions (Fig. 2D, arrowheads). These periclinal cell divisions were observed with a higher frequency in the cortex, endodermis, and epidermis compared with the wild type (Table 1). These extra cell files were observed locally and discontinued from each initial, suggesting that these defective patterns were not caused by the abnormal periclinal cell division of stem cells. In addition, PI-stained dead cells were observed with higher frequency in the epidermis, cortex, endodermis, and stele of B13.4 compared with the wild type (Fig. 2D, asterisk; Table 2).

**Table 1.** Proportions of seedlings in which extra periclinal cell divisions were observed

	Extra periclinal cell divisions (%)			
	En	Cor	Epi	*n*
Col-0	6.45	3.23	0	31
*tpr5-1*	37.1	42.9	11.4	35
*tpr5-2*	56.5	43.5	17.4	23
*tpr5-2 TPR5–GFP*	0	0	0	12

Meristem zones in 3 DAG seedlings were observed. Values are expressed as percentages of seedlings in which at least one extra periclinal cell division was observed. *tpr5-2 TPR5–GFP* is genetically identical to *tpr5-2 TPR5*(genomic)–*GFP* L1 in Fig. 4. En, endodermis; Cor, cortex; Epi, epidermis*; n*, the number of seedlings analysed.

**Table 2.** Proportions of seedlings in which dead cells were observed

	Dead cells (%)				
	En	Cor	Epi	St	*n*
Col-0	3.23	0	0	6.45	31
*tpr5-1*	2.86	31.4	62.9	74.3	35
*tpr5-2*	13.0	52.2	69.6	56.5	23
*tpr5-2 TPR5–GFP*	0	0	0	0	12

Meristem zones in 3 DAG seedlings were observed. Values are expressed as percentages of seedlings in which at least one extra periclinal cell division was observed. *tpr5-2 TPR5–GFP* is genetically identical to *tpr5-2 TPR5*(genomic)–*GFP* L1 in Fig. 4. En, endodermis; Cor, cortex; Epi, epidermis; St, Stele; *n*, the number of seedlings analysed.

### Identification of TPR5 as the causal gene for the short-root phenotype of B13.4

**Fig. 3.**
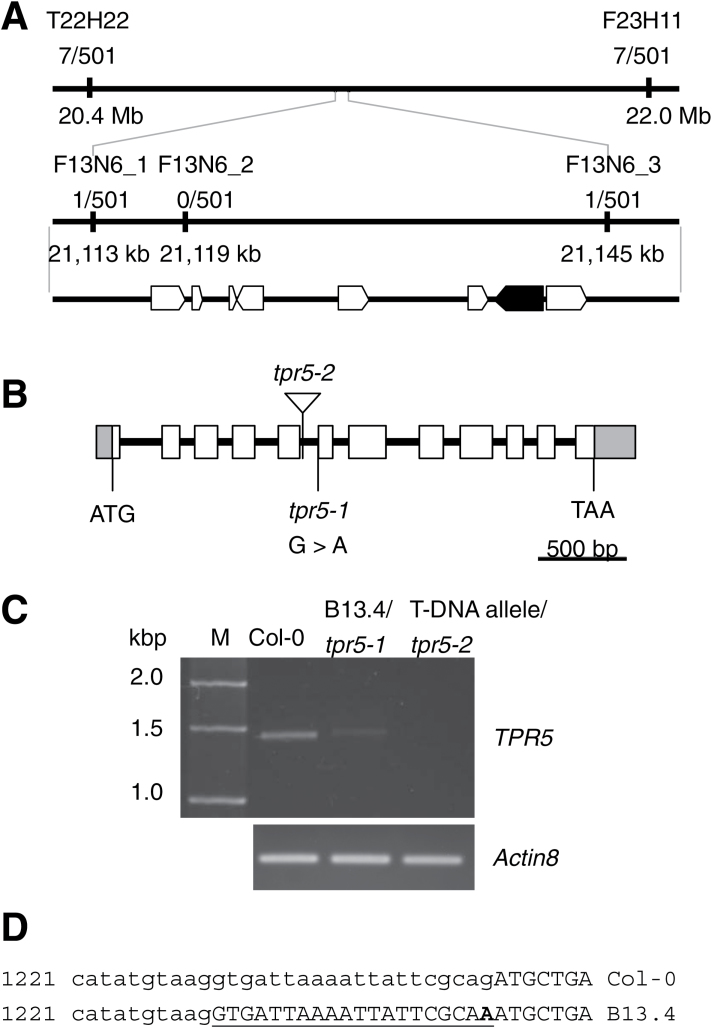
Positional identification of the mutation responsible for B13.4. Molecular markers and the number of recombinant plants found in the mapping population are shown. (A) Marker position and predicted genes in the mapped region. Eight candidate genes selected by the map-based cloning are indicated at the bottom. The gene in which a mutation was found is in black. (B) Exon–intron structure of *TPR5* and positions of mutations in *tpr5* alleles. Rectangles and bars represent exons and introns, respectively. UTRs are in grey. The T-DNA insertion is indicated by a triangle. (C)*TPR5* transcripts in *tpr5* mutants. Total RNA was prepared from whole roots of 15-d-old seedlings and the *TPR5* coding sequence and *Actin8* were amplified by RT-PCR. M, DNA size marker. (D) Altered splicing site of *TPR5* in B13.4. *TPR5* genomic sequences around the fifth intron are shown. Exons and introns are represented by upper and lower case, respectively. The numbers on the left side indicate base pair positions from the annotated transcription start site in TAIR10 annotation. The mutation in B13.4 is indicated in bold and the extra exon is underlined. Note that the extra 20bp exon causes a shift in the reading frame.

To identify the causal gene for the root growth defect in B13.4, we conducted map-based cloning. B13.4 (Col-0 background) was crossed with L*er*, and its F_2_ population was used for genetic mapping. Molecular genetic analysis of 501 individual F_2_ plants was performed using Col-0 and L*er* genetic markers and the B13.4 locus was mapped to a 32kb region on BAC clone F13N6 on chromosome 1 in a region that contained eight predicted genes (Fig. 3A). The genomic sequences of the region corresponding to the open reading frames of the eight genes were determined and only one mutation was found in *TPR5* (AT1G56440) with no mutations in the other genes. The mutation was located in the 3′ end of the fifth intron in a predicted splicing acceptor site (Fig. 3B). The nucleotide sequence of the B13.4 *TPR5* mRNA was determined by RT-PCR and sequence analysis revealed that splicing of the fifth intron of *TPR5* occurred improperly in B13.4. The *TPR5* mRNA contained an additional 20bp in the mutant (Fig. 3C, D), establishing that intact TPR5 protein was not produced in B13.4.

**Fig. 4.**
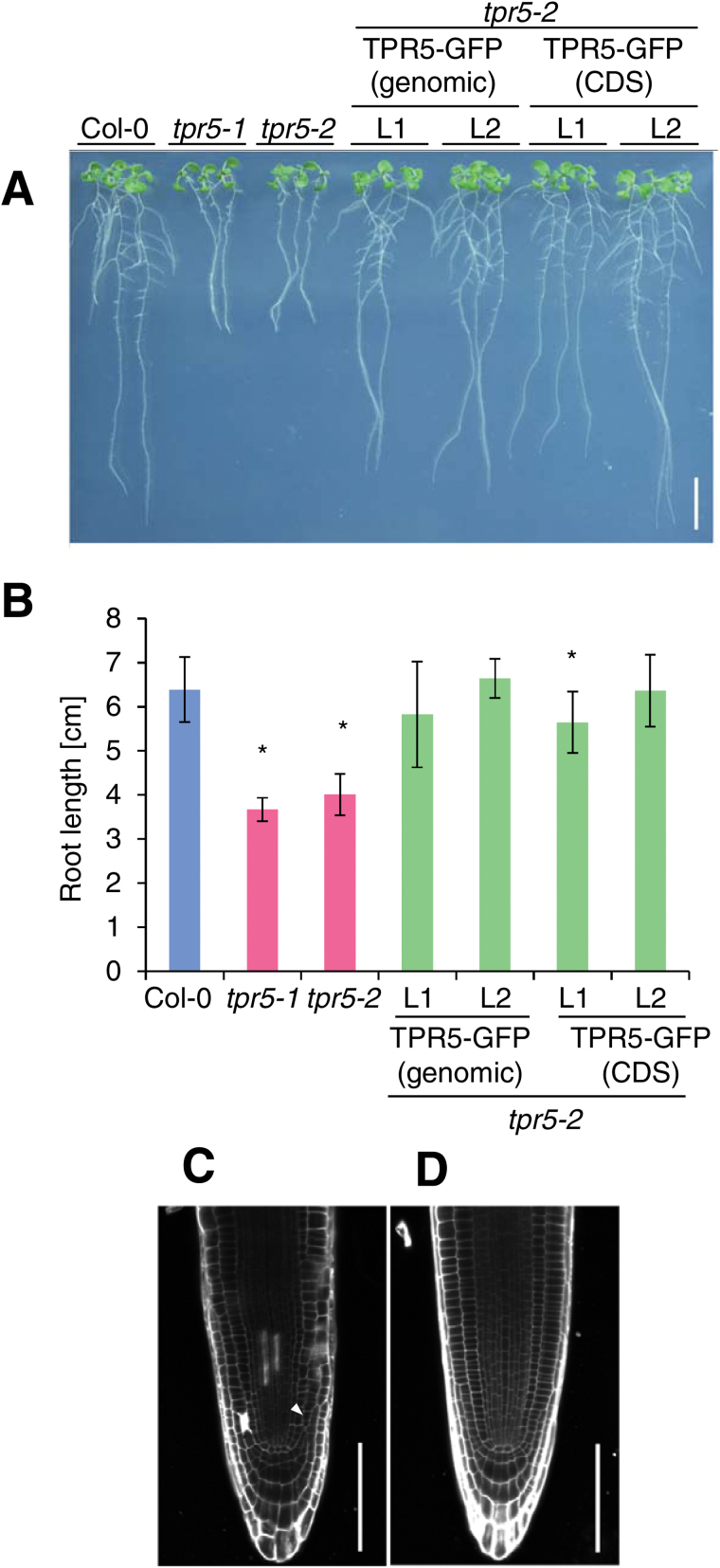
Complementation test for *tpr5-2.* (A) Root growth phenotype of *tpr5-2* complementation lines. The *tpr5-2* mutant was transformed with a GFP-fused TPR5 genomic or cDNA sequence driven by its 1.6 kbp promoter. Seven DAG seedlings are shown. Bar=1cm. (B) Root growth measurement of *tpr5* mutants and complementation lines. Primary root lengths of 7 DAG seedlings were measured. Values are means ±standard deviation of 14–19 seedlings. Asterisks indicate significant differences from Col-0 at *P* <0.05 by Welch’s *t*-test. L1 and L2 represent independent transgenic plants for each construction. (C, D) Confocal images of 3 DAG roots of *tpr5-2* (C) and *tpr5-2 TPR5* (genomic)–-*GFP* L1 (D) stained with PI. Arrowheads indicate extra periclinal cell divisions and an asterisk indicates dead cells stained with PI. Bars=100 µm.

To confirm that the mutation in *TPR5* caused the B13.4 phenotype, we obtained a tagged line allele with a T-DNA insertion in *TPR5* and isolated a line with the homozygous T-DNA insertion. We detected no intact *TPR5* transcript in the tagged line (Fig. 3C), and the allele exhibited similar defects in root growth (Fig. 4A, B) and radial organization (Fig. 4C) to B13.4. We designated B13.4 and the T-DNA allele as *tpr5-1* and *tpr5-2*, respectively. In addition, we conducted a complementation experiment by expressing a TPR5–GFP fusion protein under the control of the 5′ upstream region of *TRP5* (1.6kb upstream region from the start codon) in *tpr5-2*. We constructed two types of *TPR5–GFP* fusion proteins, one with genomic *TPR5* and the other with *TPR5* CDS. For each construct, we obtained two independent transformants homozygous for the T-DNA insertion in the T_3_ generation. All homozygous lines generated were tested for their growth and all lines recovered root elongation (Fig. 4B). In addition, root radial organization was recovered in both lines (Fig. 4C, D; Tables 1, 2).

### TPR5 promoter activity observed mainly in the stele and QC, but not in proximal meristem cells

To investigate the tissue specificity of the *TPR5* promoter activity, we generated Col-0 background transgenic plants expressing the *β-glucuronidase* (GUS) reporter gene under the control of the 1.6kb *TPR5* promoter region which was identical to the fragment used in the complementation test. We generated eight independent transformants and staining of their T_2_ generation revealed that seven of the eight lines exhibited similar staining patterns. Here we describe one of the representative transgenic plants among the seven lines.

**Fig. 5.**
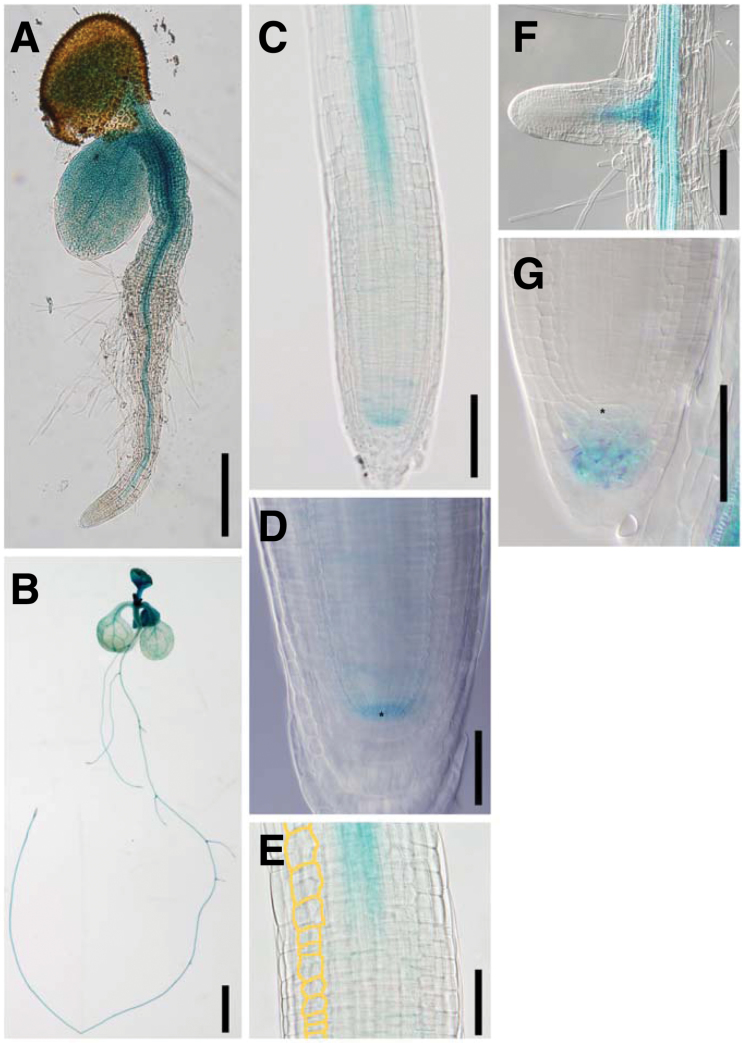
*TPR5* promoter–GUS expression patterns. Representative expression patterns of 1 DAG (A) and 8 DAG seedlings (B–G) are shown. (A) Whole seedling at 1 DAG. (B) Whole seedling at 8 DAG. (C) Primary root tip. (D) Close-up view of the meristem in the primary root. Asterisk indicates QC cell position. (E) Close-up view of elongation zone in the primary root. A cell file in the cortex is highlighted in orange. (D, F) Emerged lateral root. (G) Root tip of mature lateral root. Asterisk indicates QC cell position. Bars: (A, C, F) 100 µm; (B) 2mm; (D, E) G 50 µm.

Seedlings at 1 DAG were GUS-stained to observe the expression patterns in the early stages. GUS staining was observed in cotyledons, hypocotyls, and roots. In roots, strong staining was observed in the stele (Fig. 5A). At 8 DAG, staining was observed mainly in the vascular tissue in both roots and shoots with strong staining in young leaves (Fig. 5B). In the primary roots, GUS staining was observed mainly in the stele, QC, and several cells surrounding the QC (Fig. 5B, C, D). Weak or no GUS staining was detected in the cell division zone, other than the QC region (Fig. 5C, E) where the defect in cell division occurs in the *tpr5* mutants (Fig. 2D).

No GUS staining was observed in the tips of emerged lateral roots (Fig. 5F). On the other hand, GUS staining was observed in the columella of the elongated lateral roots, but not in the proximal meristem (Fig. 5G).

### TPR5–GFP fusion protein was localized mainly in root meristems

**Fig. 6.**
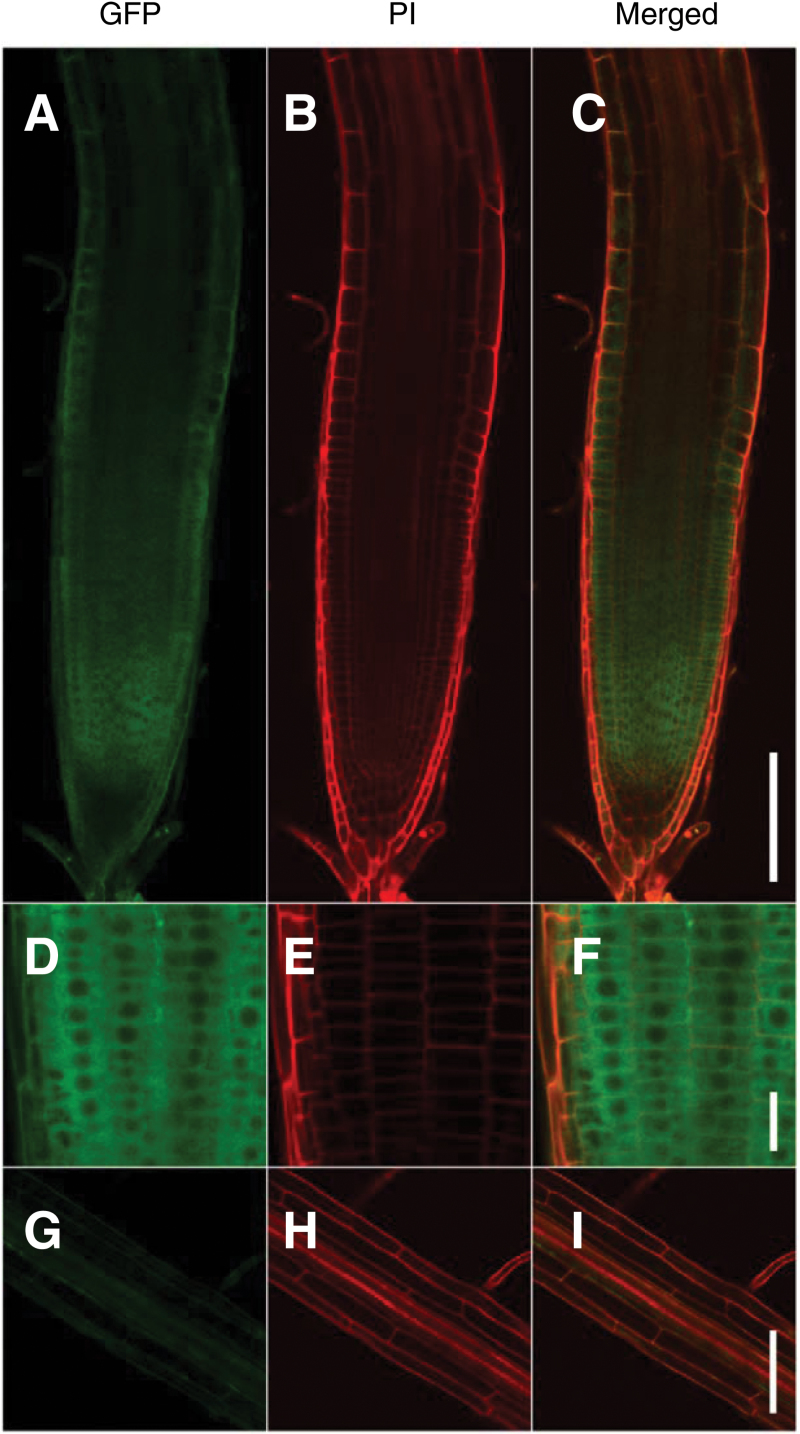
Tissue specific expression of TPR5–GFP fusion protein. Three DAG seedlings of transformants expressing TPR5–GFP (genetically identical to *tpr5-2 TPR5*(genomic)–*GFP* L1 in Fig. 4) were observed using confocal microscopy. The cell wall was stained with PI. (A–C) Representative images of primary root tips. (D–F) Primary root tips with higher magnification. (G–I) Mature region with root hair. Bars: (A–C, G–I) 100 µm; (D–F) 20 µm.

We investigated TPR5 protein localization using TPR5 (genomic)–GFP fusion in the transgenic *tpr5-2* lines used for the complementation test (Fig. 4). Driven by its own promoter, TPR5–GFP fusion showed the strongest fluorescence in the meristems in these transgenic plants which became weaker towards the elongation zone and columella cells (Fig. 6A–C). Higher magnification revealed a strong and uniform signal near the cell periphery which was absent from the central portion of the cells (Fig. 6D–F), suggesting cytoplasmic localization of the fusion protein. In mature regions of the roots, significant GFP fluorescence was not observed (Fig. 6G–I).

**Fig. 7.**
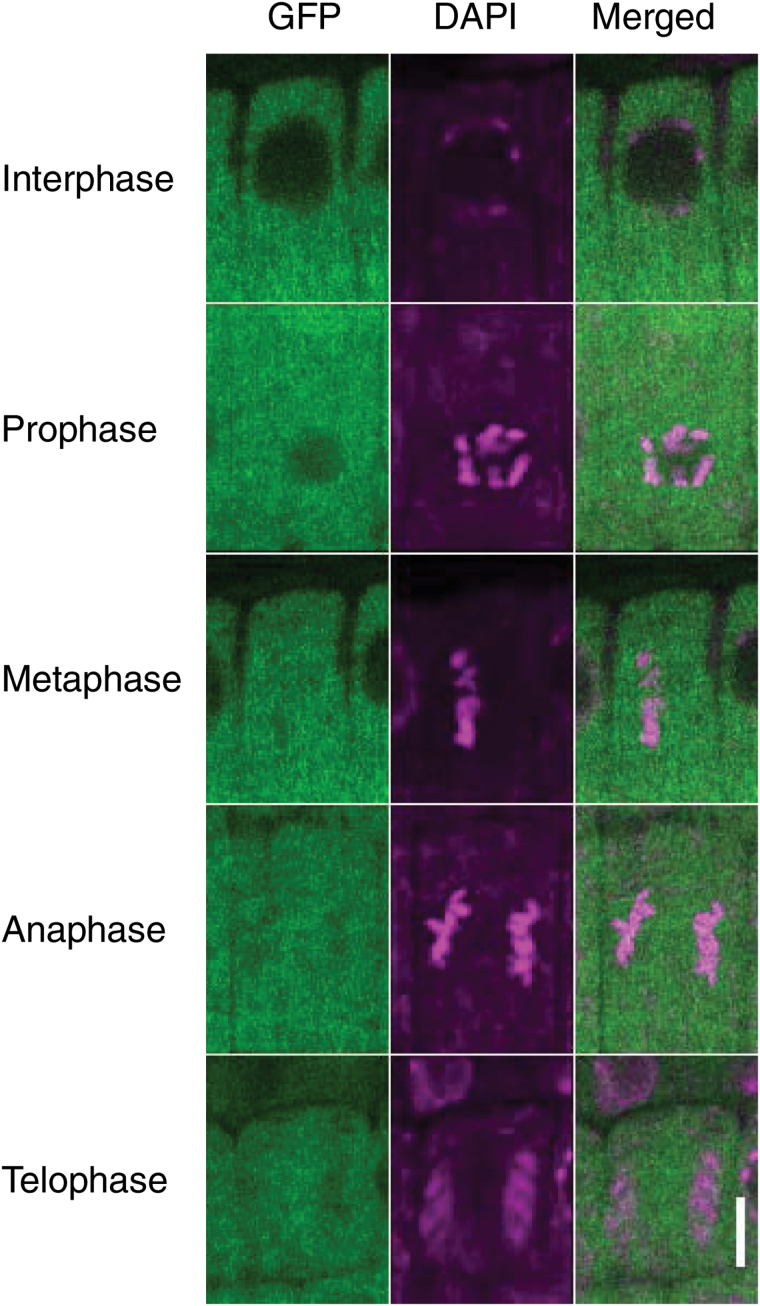
Subcellular localization of TPR5–GFP fusion protein during cell division. Three DAG seedlings of transformants expressing TPR5–GFP (genetically identical to *tpr5-2 TPR5*(genomic)–*GFP* L1 in Fig. 4) were observed using confocal microscopy. DNA was stained with DAPI and root meristematic epidermal cells in each cell division phase were observed. Bar: 5 µm.

To investigate the expression of TPR5 during each cell cycle stage further, we visualized DNA by DAPI staining and observed TPR5–GFP localization in meristematic epidermal cells (Fig. 7). During interphase and prophase of cell division, TPR5–GFP was observed in the cytosol but not inside nuclei. From metaphase to telophase, TPR5–GFP was uniformly localized in cells including the area where chromosomes were observed.

### Micronuclei were frequently observed in *tpr5* mutants

**Fig. 8.**
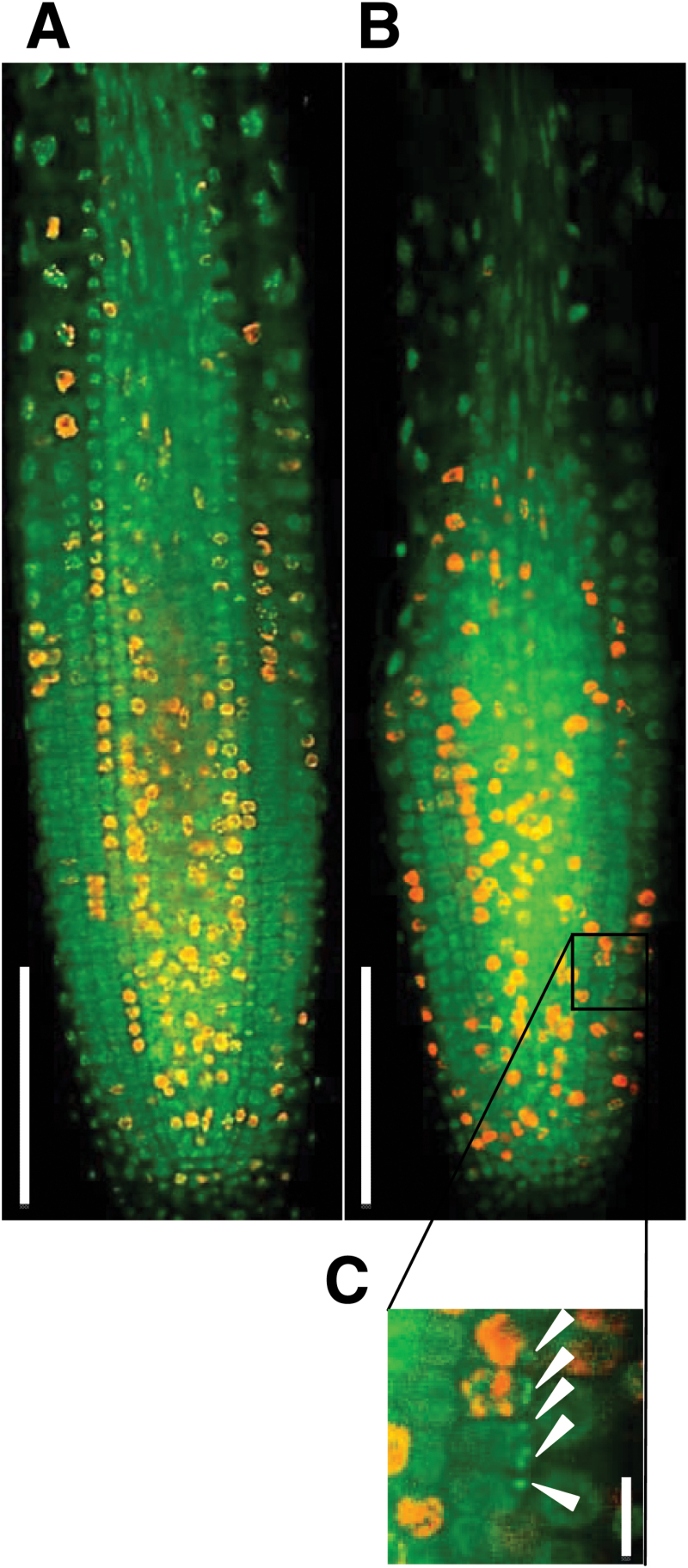
Micronucleus formation in *tpr5* mutants. In root meristems of 3 DAG seedlings, nuclei were visualized by SYBR Green I staining, and nascent DNA was labelled by pulse treatment with EdU for 30min. Representative patterns of nuclei (SYBR Green I, green) and nascent DNA (EdU, red) are shown for the wild type (A) and *tpr5-1* (B). Bars: 100 μm. (C) Magnification of a part of image (B) representing cortical cells having micronuclei (arrowheads). Bars: 10 μm.

The aberrant cell division and cell death in *tpr5* mutant root meristems motivated us to consider the possibility of cell cycle disruption. Nuclei of 3 DAG root meristems were stained with SYBR Green I (Fig. 8A, B, green signal), which revealed that 73.7% (*n*=19) of *tpr5-1* and 72.2% (*n*=18) of *tpr5-2* seedlings had at least one cell with micronuclei (Fig. 8C) in longitudinal confocal sections of root meristems, whereas no micronuclei were observed in any wild-type seedlings (*n*=17). The frequency of cortical cells with micronuclei were 0% (*n*=1380) in the wild type, 2.8% (*n*=1159) in *tpr5-1*, and 1.9% (*n*=1165) in *tpr5-2*, that suggested defects in chromosomal separation in the *tpr5* mutants. To obtain a further hint on the possible involvement of TPR5 in cell cycle progression, we visualized nuclei during DNA synthesis phase by pulse labelling using the thymidine analogue EdU and calculated the percentage of cortical cells in M or S phase in confocal sections of the root meristems. Both *tpr5-1* and *tpr5-2* exhibited slight but significantly higher proportions of mitotic cells (Supplementary Fig. S1A), whereas those in the S phase did not differ significantly between *tpr5* mutants and the wild type (Supplementary Fig. S1B). Quantitative RT-PCR revealed that *CYCB1;1* mRNA, whose accumulation is specific to the G_2_-to-M transition (Shaul *et al.*, 1996), accumulated in roots of *tpr5* mutants to a significantly higher level than in the wild type (Fig S1C). These results suggest the involvement of TPR5 in cell cycle progression.

## Discussion

### Involvement of TPR5 in root meristem maintenance through cell division

The root elongation rate was reduced in the B13.4/*tpr5-1* mutant, compared with the wild type (Fig. 1), whereas the cell length of the mature portion of the root remained unchanged (Fig. 2B). Considering the reduction in meristematic cell numbers in the B13.4/*tpr5-1* mutant, the slower root elongation in this mutant is due to reduced root meristem activity which should be explained by disturbed cell cycle progression and/or reduced number of cell cycling in the meristems. The increased proportion of cortical meristem cells in the M phase and the increased *CYCB1;1* mRNA accumulation in the *tpr5* mutants (Supplementary Fig. S1) indicate that cell cycle progression is disturbed in *tpr5* mutants. In consideration of the fact that micronuclei were observed at a higher frequency in *tpr5* mutants, TPR5 is likely to be involved in chromosomal separation since micronuclei formation is often coincident with malfunction in mitotic events, namely, a defective anaphase checkpoint, dysfunctional spindle or defects in the kinetochore (reviewed by Fenech *et al.*, 2011). The TPR5–GFP signal was observed throughout the cell cycle. During mitosis, the TPR5–GFP signal overlapped the DAPI signal representing chromosomal location (Fig. 7), suggesting that TPR5 can participate in any of those processes related to micronuclei formation. The frequently observed abnormal direction in the cell division plane in the *tpr5* mutants suggests that TPR5 is involved in the determination of the cell division plane. From these results, we conclude that *TPR5* is necessary for both activity and directionality of cell division in root meristems which is indispensable for constant cell production and maintenance of the elaborate radial structure of the root.

TPR5 was reported to harbour three TPR domains (Prasad *et al.*, 2010). The TPR domain is a protein–protein interaction domain first identified in a study of the cell cycle regulator *CDC23* (Sikorski *et al.*, 1990). Thereafter, proteins containing TPR domains were found in diverse biological processes such as transcription repression, stress response, protein kinase inhibition, mitochondrial and peroxisomal protein transport, and neurogenesis (see review by Goebl and Yanagida, 1991; D’Andrea and Regan, 2003). To our knowledge, there has been no report on the involvement of *TPR5* in any developmental processes or on its enzymatic activity.

It is well known that SCARACROW (SCR) regulates periclinal cell division in CEI daughter cells (Di Laurenzio *et al.*, 1996), which suggests *TPR5* involvement in regulation by SCR. However, ectopic expression analysis of SCR in *scr-4* revealed that activation of SCR in ground tissue induced periclinal division, but no effect was detected when SCR was expressed in other tissues, establishing that SCR acts cell-autonomously to control asymmetric cell division only within ground tissue (Heidstra *et al.*, 2004). In the case of *tpr5* mutants, cell division in an abnormal direction was not cell file-specific and not continuous from the stem cells (Table 1; Fig. 2D). Hence, it cannot be assumed that altered expression of SCR is the cause of the non-canonical periclinal cell division observed in the *tpr5* mutants. That observation also implies that TPR5 is involved in general cell division functions, rather than in the determination of cell identity.

A similar situation with *tpr5* mutants has been reported in *TONSOKU/MGOUN3/BRUSHY1* mutants, which is involved in the stabilization of chromatin structure (Guyomarc’h *et al.*, 2004; Suzuki *et al.*, 2004; Takeda *et al.*, 2004). Those mutants exhibit root growth defects and oblique cell division in root meristems, although the length of fully expanded cells is comparable to that of the wild type. Those mutants accumulate cells expressing *CYCB1;1:GUS* in shoot and root apical meristems, suggesting that cell cycle progression at the G2/M phase is important for regulating cell division patterns during plant development (Suzuki *et al.*, 2005; Inagaki *et al.*, 2006). It was reported that *TONSOKU/MGOUN3/BRUSHY1* interacts with TSK-associating protein 1 (TSA1) through the LGN motif which is categorized as a TPR motif subfamily and their involvement in mitosis has been suggested (Suzuki *et al.*, 2004). As in *TONSOKU/MGOUN3/BRUSHY1*, it is likely that TPR5 participates in some protein complexes that function in cell division. Identification of the interactors is a further subject of study.

### Tissue specificity of TPR5 expression

The *TPR5* promoter activity was observed mainly in steles and around QCs but not in proximal meristems (Fig. 5). By contrast, fluorescence of TPR5–GFP fusion protein was observed in whole root meristems, including the region where extra periclinal cell division and cell death were observed (Fig. 6). It is possible that the coding region of *TPR5* harbours elements essential for expression in the meristem or there might be a non-cell-autonomous function via movement of *TPR5* mRNA or protein. Although the mechanisms for the discrepancy between tissues with promoter activity and the GFP fusion protein remain unknown, we can conclude that the TPR5 protein is expressed and functions in root meristems judging from the spatial concordance between localization of the fusion protein and observed meristem phenotype.

## Supplementary data

Supplementary data can be found at *JXB* online.

Table S1. Primers used in this study.

Table S2. Genetic markers near the *tpr5-1* mutation used in map-based cloning.

Fig. S1. Proportions of the cortical cells in M or S phase in *tpr5* mutants.

Supplementary Data

## Abbreviations:

CDS: coding DNA sequence
CEI: cortex/endodermis initials
DAG: day after germination
DAPI: 4′,6-diamidino-2-phenylindole
EdU: 5-ethynyl-2′-deoxyuridine
GFP: green fluorescent protein
GUS: β-glucuronidase
PI: propidium iodide
PPB: pre-prophase band
TPR: tetratricopeptide repeat
QC: quiescent centre
RT-PCR: reverse transcription-PCR
SNP: single nucleotide polymorphism
SSLP: single sequence length polymorphism.
