# Supplementary material for: TPR5 is involved in directional cell division and is essential for the maintenance of meristem cell organization in Arabidopsis thaliana
Source: J Exp Bot. 2016 Feb 17;67(8):2401–11. doi: 10.1093/jxb/erw043 (PMC4809291; doi:10.1093/jxb/erw043)
Supplement: Supplementary Data [file supp_67_8_2401__index.html]

 TPR5 is involved in directional cell division and is essential for the maintenance of meristem cell organization in Arabidopsis thaliana — TPR5 is involved in directional cell division and is essential for the maintenance of meristem cell organization in Arabidopsis thaliana — Supplementary Data 

# *TPR5* is involved in directional cell division and is essential for the maintenance of meristem cell organization in *Arabidopsis thaliana*

## Supplementary Data

Data files

- supplementary\_table\_S1\_S2\_figure\_S1.pdf - Supplementary Data
