# Supplementary material for: TPR5 is involved in directional cell division and is essential for the maintenance of meristem cell organization in Arabidopsis thaliana
Source: J Exp Bot. 2016 Feb 17;67(8):2401–11. doi: 10.1093/jxb/erw043 (PMC4809291; doi:10.1093/jxb/erw043)
Supplement: Supplementary Data [file supp_erw043_supplementary_table_S1_S2_figure_S1.pdf]

Supplementary materials

Title
TPR5 is involved in cell cycle progression and is essential for the maintenance of meristem cell organisation in *Arabidopsis thaliana*

Authors
Naoyuki Sotta, Lukram Shantikumar, Takuya Sakamoto, Sachihiro Matsunaga and Toru Fujiwara

Table S1. Primers used in this study

Name	Sequence
TPR5_pro_F	CACCATGGATGGCACACAGGGAAC
TPR5_CDS_R	CTGTTTAAGGCAGTATCTTGAC
TPR5_pro_R	TCTGAGAGAGATTCTCCGGCGAGC
TPR5_CDS_F	CACCATGGCTAGGTCACCGAGCAAAC
TPR5_fuse_F	CGGAGAATCTCTCTCAAATGGCTAGGTCACCGAGC
TPR5_fuse_R	GCTCGGTGACCTAGCCATTCTGAGAGAGATTCTCCG
Actin8_RT_F	GCCAGATCTTCATCGTCGTG
Actin8_RT_R	TCTCCAGCGAATCCAACCTT
CYCB1;1_RT_F	TAAGCAGATTCAGTTCCGGTCAAC
CYCB1;1_RT_R	GGGAGCTTTACGAAAGAAATACTCC
SALK099949_LP	CATTTCTGTCAGAAGGCTTCG
SALK099949_RP	CAGAAGAAGTTCAATGAGGCG

Table S2. Genetic markers near the *tpr5-1* mutation used in map-based cloning

Maker	Forward	Reverse	Type
T22H22	TTTATCAGCTCCTGCATGCTT	AAAAGAAGAGAGGGAGGCTCA	SSLP
F13N6_1	CATACCTCGACGCACAGCTA	TTCATTTCATCCATCGCAGA	SNP
F13N6_2	GAAGCTGGACGAGGATGAAG	AGCGGGAAAATAAAGCAGGT	SSLP
F13N6_3	TTGTTGGGATTGGGAAAAAC	CGGGATAACGCGATTTAGTG	SSLP
F23H11	GGTTTGATGGAGATTTTGCTG	GGGATATTGCTGCAAAGGAA	SSLP

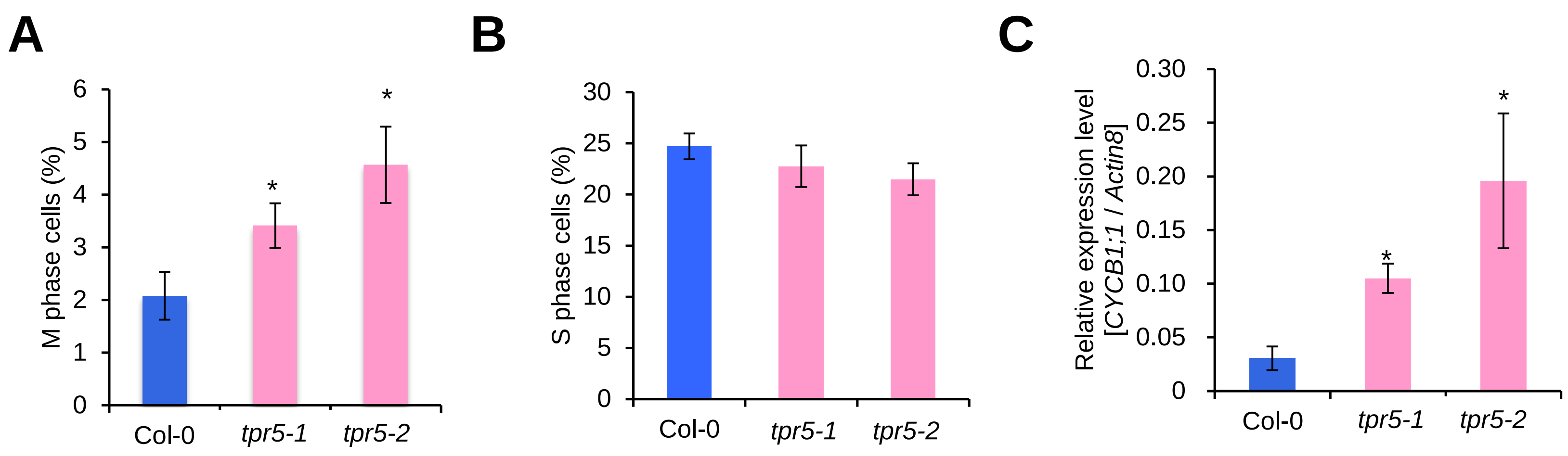

Fig. S1. Proportions of the cortical cells in M or S phase in *tpr5* mutants
(A) Proportion of cortex cells showing any mitotic features (prophase, metaphase, anaphase and telophase). Asterisks indicate a significant difference from Col-0 at p < 0.05 by Welch's *t*-test. (B) Proportion of S phase cells in the cortex. Values in (A) and (B) are the mean ± standard errors of 17–19 seedlings. There was no significant difference between wild type and each mutant at *p*> 0.05 by Welch's *t*-test. (C) mRNA accumulation of CYCB1;1 in *tpr5* mutants. mRNA was extracted from roots of 15 day-old seedlings and *CYCB1;1* mRNA accumulation was determined by qRT-PCR. Expression levels are normalized by that of *Action8* and expressed in the mean ± standard errors of three biological replication. Asterisks indicate a significant difference from Col-0 at *p* < 0.05 by Welch's *t*-test.

Sotta et al., 2015
Fig. S1
